# Letter to the Editor Re: Billeaud et al. *Nutrients* 2018, *10*, 690

**DOI:** 10.3390/nu11010103

**Published:** 2019-01-06

**Authors:** Wolfgang Bernhard, Christian F. Poets, Axel R. Franz

**Affiliations:** 1 Department of Neonatology, Children’s Hospital, Eberhard Karls University, 72076 Tübingen, Germany; christian-f.poets@med.uni-tuebingen.de (C.F.P.); axel.franz@med.uni-tuebingen.de (A.R.F.); 2 Center for Pediatric Clinical Studies, Children’s Hospital, Faculty of Medicine, Eberhard-Karls-University, 72076 Tübingen, Germany

**Keywords:** arachidonic acid, docosahexaenoic acid, linoleic acid, preterm infant nutrition, milk supplements, breast milk

**Dear Editor,**

Billeaud and co-authors recently described the effects of a new middle-chain fatty acid and docosahexaenoic acid enriched breast milk fortifier to improve preterm infants’ lipid nutrition [1]. This is a commendable task. However, the encouraging statements in the introduction, emphasizing the importance of both arachidonic (ARA) and docosahexaenoic acid (DHA), for preterm infant development, aren’t mirrored by the composition of the fortifier and the results presented. First, the fortifier is not comprised of substantial amounts of ARA and its ARA/DHA ratio is <1, whereas the physiological ARA/DHA ratio would be >1 (Table 1). 

Moreover, previous data shows that preterm infants’ main plasma phospholipid, phosphatidylcholine (PC), is dominated by PC comprising large and postnatally increasing amounts of linoleic acid (LA, C18:2-n-6) [2,3]. Approximately 40–45% of PC species have an LA residue, ~22% an oleic acid (OA, C18:1n-9), ~20% an ARA (C20:4n-6) and ~6% a DHA (C22:6n-3) residue. This is in contrast to the fetal situation, where ARA-PC surmounts LA-PC by far, and DHA-PC is higher as well [3]. As these PC subgroups contain a second, mostly saturated fatty acid, the PC fatty acid composition in preterm infants equals 20–22% LA, 11% OA, 10% ARA and 3% DHA. The data by Billeaud et al., however, shows a rather different composition, with only ~3% LA, 15% OA, ~1–2% ARA and ~1% DHA in plasma phospholipids (Table 4). Similarly, the LA values in plasma triglycerides are surprisingly low, underestimating the postnatal increase and critical role of high LA in preterm infant plasma lipids, possibly competing with ARA and DHA homeostasis.

Finally, the authors claim to improve our insight into metabolism. The latter, however, is best assessed via isotope labelling and the investigation of fluxes and kinetics rather than fatty acid profile comparisons. The authors write “One can therefore hypothesize that supplying dietary lipids to stimulate the formation of monounsaturated fatty acids, and in particular n-9 long-chain monounsaturated fatty acids, during the neonatal period is an appealing nutritional strategy to support the myelination process in the preterm infant”. This is well said, but such an increased formation is not backed by their data shown in Tables 4–7, rather showing constant values of OA (C18:1n-9) in lipid fractions (Table 5). 

In essence, there is no indication that this new human milk fortifier may correct for the principle disturbance of preterm infant fatty acid metabolism compared to the fetus, which is characterized by impaired ARA and DHA supply and homeostasis, and LA oversupply, although this is an urgent task.
